# Batch-Level Average Market Weight Estimation and Interpretability Analysis for Pigs with Regional K-Means Clustering and AMFormer

**DOI:** 10.3390/ani16132092

**Published:** 2026-07-06

**Authors:** Yan Chen, Ruiwen Liu, Yanbin Liang, Hongxing Peng, Guangmin Huang

**Affiliations:** 1 College of Mathematics and Informatics, South China Agricultural University, Guangzhou 510642, China; cheny@scau.edu.cn (Y.C.); xyphx@scau.edu.cn (H.P.); ptmkpf@163.com (G.H.); 2 Wens Foodstuff Group Co., Ltd., Xinxing, Yunfu 527400, China; 13826886700@139.com

**Keywords:** deep learning, pig weight estimation, tabular data, K-means clustering, regression network

## Abstract

In modern pig farming, batch-level weight estimation is important for optimizing feeding management and improving economic efficiency. Current vision-based weight estimation methods are often affected by acquisition conditions and mainly focus on individual pigs, making them less suitable for batch-level decision-making. To address this problem, this study focuses on concurrent estimation of batch-level final average market weight and explores the use of tabular production data for non-invasive body weight estimation in pig groups. Based on more than 35,000 production records from a modern pig farming enterprise, we propose RC-AMFormer. The method uses region-wise K-means clustering to characterize underlying farming heterogeneity, introduces cyclic month encoding and organizational hierarchy features, and adopts the arithmetic attention mechanism in AMFormer to model feature interactions. Among the ten comparison models, RC-AMFormer achieves an MAE of 2.64, an MSE of 11.77, and an $R^{2}$ of 0.67 on the test set. Compared with XGBoost, it reduces MSE and MAE by 4.03% and 1.43%, respectively. SHAP analysis identifies important features associated with market weight. These results suggest that tabular production data provide a feasible basis for batch-level non-invasive body weight estimation and may also support similar production scenarios.

## Simple Summary

Estimating pig market weight is important for production management and economic efficiency in modern pig farming. In this study, more than 35,000 batch-level production management records are used to estimate final average market weight. Based on practical farming characteristics, we combine regional clustering with a deep learning method for tabular data. The proposed method achieves an average error of about 2.6 kg and shows competitive performance compared with ten strong baseline models. In addition, features associated with market weight are also analyzed. These findings suggest that tabular production data may provide a feasible basis for batch-level non-invasive body weight estimation and contribute to more precise pig farming.

## 1. Introduction

In modern intensive pig production, body weight is an important indicator of growth performance, health status, and production efficiency, and it provides an important basis for production management decisions [1]. Accurate body weight information is essential for feeding management, production scheduling, marketing planning, and sales decisions. Therefore, pig weight estimation has become a basic task in smart pig farming [2].

Traditional pig weight estimation methods mainly rely on manual weighing or body-size measurements [3]. However, these methods are time-consuming, labor-intensive, and inefficient and may also cause unnecessary stress to pigs [4]. In recent years, computer-vision-based non-invasive weight estimation methods, including those based on 2D images and 3D point clouds, have become an important direction in pig weight estimation [5,6,7]. Existing studies have achieved high estimation accuracy at the individual-pig level by extracting body-shape and body-size features, such as body length, body width, and contour area, as well as geometric features from point clouds [8,9,10].

Although vision-based weight estimation has improved the level of automation in pig weight measurement, its practical application still faces several limitations. On the one hand, the performance of these methods is often affected by the data acquisition environment, occlusion, lighting conditions, and device deployment [5]. On the other hand, most existing studies focus on individual pigs, while relatively little attention has been paid to weight estimation at the group or batch level. In practical production management, customer matching, production scheduling, and sales decisions are usually conducted at the batch level rather than for individual pigs [11]. Therefore, individual-level estimation results are often insufficient to fully support practical business needs.

For commercial pig production, market weight estimation is also directly related to carcass grade and sales revenue. According to the Chinese national standard Code of Practice for Quality Grading of Livestock and Poultry Meat (GB/T 40945-2021) [12], commercial pigs are graded and priced based on carcass weight. However, carcass weight cannot be directly measured before marketing. Since carcass weight has a stable relationship with live market weight [13,14], accurate estimation of batch-level market weight can help farming enterprises plan batch sorting and packaging, match products to target customers, and implement grade-based sales strategies [1,2].

With the development of large-scale and standardized pig production systems [15,16,17], modern pig farming enterprises have widely established relatively complete production management information systems. These systems have accumulated large amounts of structured production data related to pig farming [18,19], which have been used in scenarios such as hog price forecasting, production cycle management, and production optimization [11,20,21]. Production management data are derived from routine records in enterprise production systems and do not require additional hardware for data acquisition or direct measurement of pigs. Therefore, using such data for pig group weight estimation and batch-level market weight estimation provides a new research direction for non-invasive body weight estimation in pig groups. Compared with vision-based weight estimation methods, this approach does not require additional image acquisition devices or direct contact with animals and thus has lower data acquisition costs and potential for broader application.

Since production management data are usually stored in structured tabular form, estimating pig group weight based on such data can be naturally formulated as a tabular regression task. In existing weight estimation studies, gradient-boosted decision tree (GBDT) models, such as XGBoost [22], CatBoost [23], and LightGBM [24], are commonly used in the regression stage. Due to their strong performance and robustness, GBDT models have long dominated the field of tabular learning [25,26,27]. However, tree-based models still depend to some extent on the quality of input features, and their ability to model high-order feature interactions is relatively limited [28].

In recent years, following the success of deep learning in natural language processing [29] and computer vision [30], researchers have increasingly introduced deep learning into tabular data modeling. These methods have also been widely applied in key fields such as agriculture, finance, and healthcare [31,32,33,34,35]. Compared with traditional machine learning methods, deep tabular learning shows stronger representation ability in large-scale, high-cardinality, and transfer learning scenarios [26,36]. Among these methods, Transformer-based tabular models can effectively capture complex feature interactions through self-attention and have achieved competitive performance [26]. Representative models include AutoInt [37], SAINT [38], FT-Transformer [39], ExcelFormer [40], AMFormer [41], and MAYA [42]. These advances provide a new technical path for pig group weight estimation and batch-level market weight estimation based on tabular data.

However, most existing tabular learning methods are designed for general-purpose settings and usually treat samples as a homogeneous whole, which may overlook relevant prior information in practical scenarios. Even within a standardized production system, production performance may vary across farms because of variations in regional conditions, organizational management, seasonal climate, and other factors. Therefore, how to incorporate such prior knowledge into the model is an important issue for batch-level market weight estimation.

Based on this, we propose RC-AMFormer, a method for non-invasive concurrent estimation of batch-level final average market weight using tabular production data derived from enterprise production systems. The method adopts AMFormer [41] as the backbone for tabular feature modeling and uses K-means clustering [43] to capture underlying heterogeneity in large-scale standardized pig production. Cyclic encoding is further applied to time-related features to capture seasonal climate effects, and management-related hierarchical features are introduced to enhance the model representation. Experimental results on more than 35,000 real-world production records show that RC-AMFormer achieves better overall performance than 10 baselines, including XGBoost and AMFormer. Finally, SHAP analysis is conducted to identify important factors associated with market weight estimation and provide some interpretability for the model results.

## 2. Materials and Methods

### 2.1. Data

#### 2.1.1. Batch Production Records of Pigs

The production records used in this study were collected from modern pig farms of Wens Foodstuff Group (hereinafter referred to as “Wens”), whose farming operations cover five major regions of China, namely East China, South China, Central China, North China, and Southwest. Wens adopts an enterprise–farmer model, under which the company provides piglets, feed, animal health services, technical support, and hog procurement, while farmers are responsible for daily feeding and management during the fattening stage. To ensure production quality, Wens has established a standardized management system and digitally manages the farming processes of participating farmers. Under this system, the production process of each batch of pigs raised by farmers is recorded throughout the fattening stage, resulting in structured production records. The current empirical study focuses on production batches of commercial pigs under a standardized fattening system.

The data contain 36,536 independent batch records. Each sample (i.e., each row) represents one production batch of pigs, and each feature (i.e., each column) records a production indicator. In total, the data include 13 features and 1 target variable, which can be grouped into four categories.

- Organizational and regional indicators: The l2 unit, l3 company, l4 company, and l5 unit indicate the affiliation path of each farm within the enterprise’s multi-level management system. The Region feature describes the geographical location of the farm.
- Initial batch conditions: Placement month, placement count, and placement weight. These indicators characterize the initial status of each batch.
- Production process indicators: Feeding days, 2w mortality, 5w mortality, medication cost, and feed consumption. These indicators reflect pig status and cost input during the feeding process.
- Marketing outcome indicator: market weight. This variable serves as the estimation target in this study.

The details of the data are shown in Table 1.

#### 2.1.2. Production Phase and Data Collection

The data used in this study cover the production cycle from piglet placement to marketing at contract farms. Piglets are nursed at the company’s sow farms for 23–25 days, reaching a body weight of 6–7 kg, and are transferred to contract farms within 1–3 days after weaning. Upon arrival, they are assigned to pens, and information such as placement month and placement weight is recorded. During the subsequent fattening stage, relevant production management data continue to be recorded. Pigs are marketed when they reach the enterprise’s market standards.

#### 2.1.3. Indicator Definitions and Data Sources

This section further explains the definitions and data sources of several key indicators.

**Figure 1:**
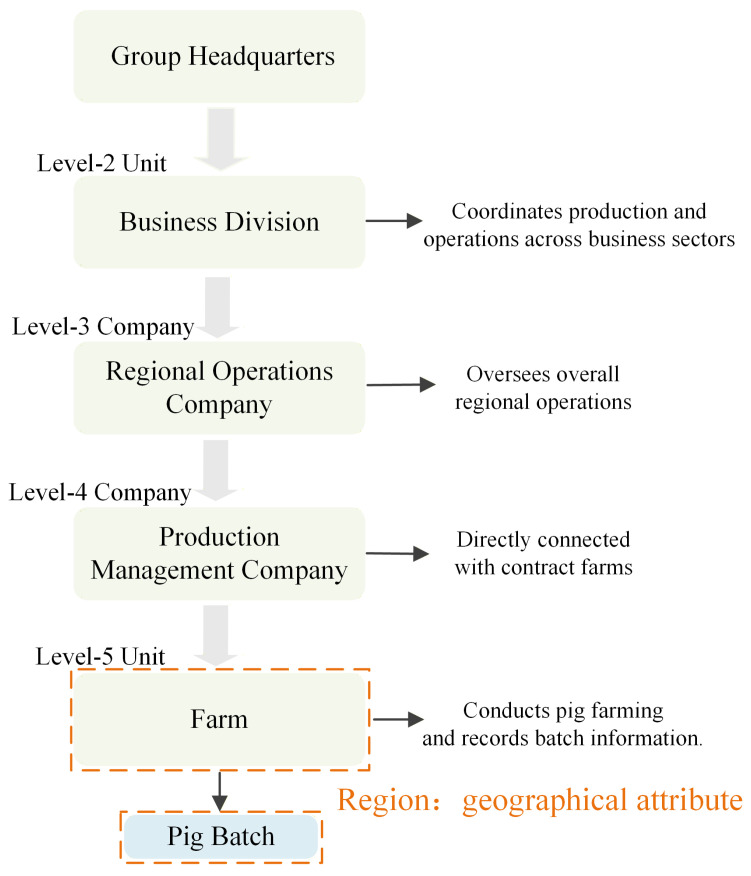
Organizational management hierarchy.

- Feed Consumption. In actual production, the enterprise delivers piglets and feed to contract farmers and records the placement count and cumulative feed supply for each production batch through the production management system. Feed is supplied on farms through automatic feeding lines. However, the feed consumption per pig used in this study is not individual or group-level feed intake monitoring data. Instead, it is a production indicator calculated by the company headquarters based on the cumulative feed supply recorded in the production database and the placement count, which is calculated as follows:
  (1) $$\mathrm{Feed}\;\mathrm{Consumption}=\frac{\mathrm{Cumulative}\;\mathrm{Feed}\;\mathrm{Supply}}{\mathrm{Placement}\;\mathrm{Count}}$$
  where CumulativeFeedSupply represents the cumulative feed delivered to the batch over the entire feeding period, as recorded by the enterprise’s management system.
- Feeding Days. This indicator denotes the cumulative number of days that pigs in a batch have been raised since piglet placement. It is used to characterize the growth stage and farming progress of the batch.
- Organizational hierarchy feature. Wens has established a relatively complete multi-level organizational management structure. The organizational hierarchy features used in this study correspond to management companies or units at different granularities below the group headquarters. Specifically, this hierarchy is structured as follows, headquarters → business division (l2 unit) → regional company (l3 company) → production company (l4 company) → farm (l5 unit).
  As shown in Figure 1, under large-scale standardized pig production, each production batch can be traced through this hierarchy to obtain its complete management affiliation path. In addition, each batch is labeled with its geographical region, including South China, East China, North China, Central China, and Southwest. The Region feature mainly reflects the macro-geographical location of the batch and its possible climatic and environmental differences. It is not part of the enterprise’s internal organizational hierarchy.

**Figure 2:**
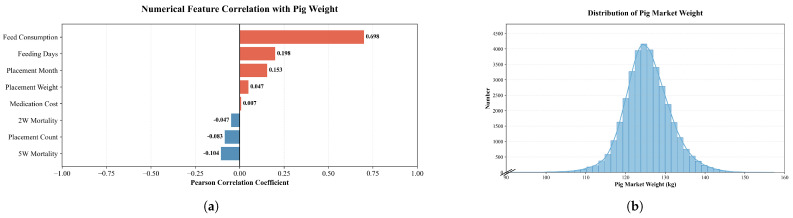
Descriptive analysis of the data. (**a**) Pearson correlation coefficients of numerical features. (**b**) Distribution of average market weight.

These production records form batch-level pig production data. Among these indicators, feed consumption per pig is a key indicator of nutritional input and is closely associated with market weight [44]. Placement weight, feeding days, and mortality-related indicators further reflect pig growth status and management conditions from different perspectives. Figure 2a,b present the Pearson correlation coefficients of the cleaned numerical features and the distribution of market weight, respectively. As shown in Figure 2b, the target variable follows an approximately normal distribution, indicating that it is suitable for regression modeling.

#### 2.1.4. Data Cleaning

We clean the raw pig production records through two stages, namely rule-based screening and statistical screening. The rule-based screening rules are summarized in Table 2. In this stage, relatively conservative thresholds are applied to remove clearly unreasonable values. This setting is used because some indicators are manually recorded by contract farmers, whose digital literacy may vary [45]. Therefore, this stage mainly aims to exclude obvious data entry errors in farmer-reported records and values that violate business logic.

For the samples that pass the rule-based screening but may still exhibit abnormal patterns in the data distribution, we further apply statistical methods for identification. Specifically, Isolation Forest [46], an unsupervised method that identifies anomalies through random partitioning, is used to detect anomalies not captured by explicit rules. The detected anomalies in numerical variables related to growth, cost, and mortality are then validated using the Z-score method, which measures the degree to which a value deviates from the mean and is calculated as follows:

(2) $$Z=\frac{X-\mu }{\sigma }$$

Samples with |Z| > 3 are classified as statistical outliers. To further reduce the false positive rate, a sample is classified as a statistical anomaly only if it is identified by both Isolation Forest and the Z-score method. Finally, we remove 690 samples identified by the rule-based criteria and 355 statistical anomalies from the data. The remaining 35,491 valid records are then used to construct a batch-level pig production dataset for subsequent modeling.

### 2.2. Problem Definition

This study aims to estimate the final average market weight of each batch based on production management records. We denote the batch production records as $X=\{x_{1},x_{2},\ldots ,x_{n}\}$, where *n* means the total number of samples, $x_{i}\in R^{k}$ denotes the specific indicators of the *i*-th sample, and k=13 denotes the number of original features. The target values are denoted as $Y=\{y_{1},y_{2},\ldots ,y_{n}\}$, where $y_{i}$ is the actual average market weight of sample $x_{i}$. Based on the training data *X* and *Y*, a regression function f(X;Y;θ) with trainable parameters θ is learned to estimate market weight from input features. Given a new batch sample, the learned function outputs its estimated value.

### 2.3. Weight Estimation Model

**Figure 3:**
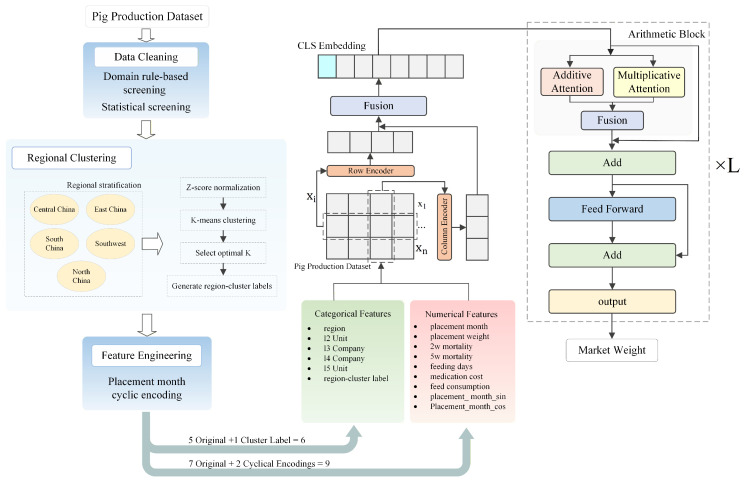
The framework of RC-AMFormer. The method includes data cleaning, regional clustering, and feature engineering. This is followed by the row–column fusion architecture, the arithmetic attention network from the original AMFormer, and the regression output layer. The model takes 6 categorical features and 9 numerical features as input and outputs the estimated value.

In this section, we introduce RC-AMFormer, a method designed for batch-level average pig market weight estimation. In standardized pig farming, production batches may still exhibit latent heterogeneity and may also be affected by seasonal climate factors. The method builds upon AMFormer [41] by introducing regional clustering, row–column fusion embedding, and cyclic encoding. The overall framework is shown in Figure 3.

#### 2.3.1. Regional Clustering Method

Under the large-scale enterprise–farmer model, pig farms are managed through a multi-level organizational structure. In our dataset, the management units exhibit a clear hierarchy, including the l2 unit, l3 company, l4 company, and l5 unit. Different organizational levels correspond to different granularities of management and execution and may therefore lead to different degrees of production variation.

**Figure 4:**
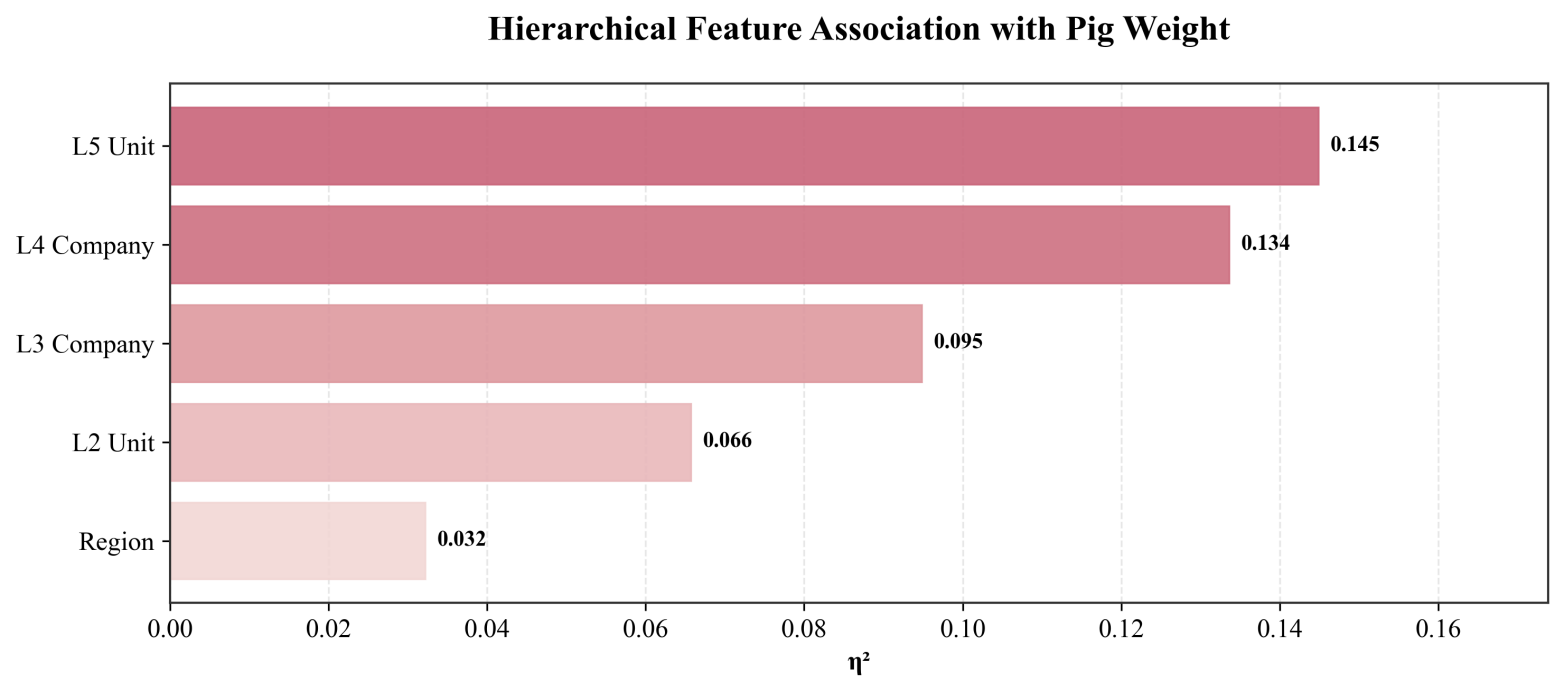
Effect sizes of geographical and hierarchical features.

As shown in Figure 4, the effect size analysis of hierarchical features indicates that the explanatory power of organizational hierarchy for market weight increases significantly as the management granularity becomes finer. Among all levels, the l5 unit, which is directly responsible for frontline production guidance, has the strongest explanatory power for market weight. This result suggests that market weight is closely related to fine-grained differences at the farm level, and farm-level conditions and farmers’ actual management practices may be important contributing factors.

At the same time, although production inputs and feeding processes are highly standardized in large-scale pig production, substantial intrinsic heterogeneity still commonly exists across samples due to differences in geographical environment, climatic conditions, management level, and execution at the farm level. If all data are modeled as following a homogeneous distribution, this important prior information may be overlooked. Based on this, we design a regional clustering method to characterize the fine-grained differences that remain within each region under the standardized production framework and to transform them into structured features that can be utilized by the model.

Common clustering methods include density-based clustering, partition-based clustering, hierarchical clustering, and deep clustering [47]. Considering that the features used for clustering in this study are all continuous numerical variables, and that the overall data distribution is relatively regular, with no significant outliers or obvious density imbalance, the partition-based K-means [43] is adopted for clustering analysis. As a classical unsupervised learning method, K-means uses Euclidean distance as the distance measure and offers high computational efficiency, stable results, and good interpretability.

Specifically, the dataset is stratified into five regions, namely South China, East China, North China, Central China, and Southwest. Candidate indicators are analyzed and screened based on whether they can reflect differences in the farming process while remaining interpretable. According to this principle, four numerical indicators are selected for clustering, namely feed consumption, 2w mortality, medication cost, and feeding days. These indicators mainly reflect four aspects, namely feed input, farm conditions and execution, health, and production cycle.

In addition, 2w mortality is used instead of 5w mortality to introduce early mortality information that is closer to the initial post-placement period into the clustering features. The first two weeks correspond to a stage in which management interventions, including piglet transfer, feed transition, and grouping management [48], are relatively intensive. Mortality and culling during this period may be more closely related to management quality. In comparison, the 5w mortality variable covers a longer observation window and may also include information related to later health status, disease occurrence, treatment intervention, and environmental changes [49].

After splitting the dataset, we perform clustering separately within each region using the training samples. Specifically, for the clustering features within each region, we first apply Z-score standardization to the training set to avoid bias caused by different feature scales. The standardized values are then grouped by the K-means. Candidate cluster numbers K∈{2,3,4} are examined to determine the value of *K* that maximizes the Silhouette Coefficient for each region. In a standardized production system, actual farming differences within a region are usually relatively limited and interpretable and can generally be summarized into two to four typical types. If five or more clusters are used, the model is more likely to overfit data noise. When K=1, heterogeneity in the farming process is completely ignored. Therefore, the value of *K* that maximizes the Silhouette Coefficient is selected independently to balance interpretability and model stability. Differences in the final number of clusters across regions also reflect different degrees of farming variation under the standardized production framework.

To verify the stability of the regional clustering results, we fix the optimal number of clusters for each region, repeat K-means 30 times with different random seeds, and evaluate the clustering consistency using the Adjusted Rand Index (ARI) [50]. As shown in Table 3, the mean ARI is above 0.90 in all regions. In addition, the relatively small standard deviations, ranging from 0.0038 to 0.0200, also indicate that the regional clustering results are reasonably stable.

**Figure 5:**
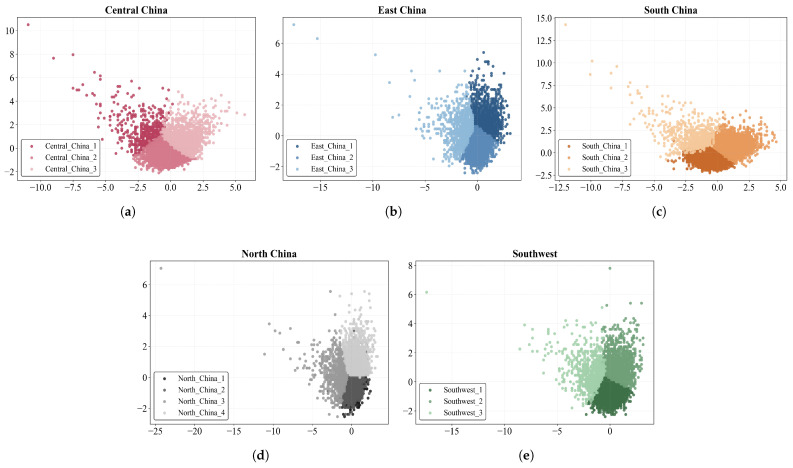
PCA visualization of K-means clustering results. (**a**) Central China. (**b**) East China. (**c**) South China. (**d**) North China. (**e**) Southwest.

After clustering, samples with similar values across the four indicators are assigned to the same cluster, thereby identifying several relatively homogeneous farming subgroups within each region. Subsequently, the region-specific clustering models fitted on the training set are used to assign cluster labels to the validation and test samples. Finally, each batch is assigned a joint region–cluster label, such as “South_China_0” or “East_China_2”. To intuitively show the regional clustering structure, we further apply PCA to the selected numerical features and visualize the two-dimensional distribution of all samples, as shown in Figure 5. The resulting joint discrete label is then used as an additional categorical feature and fed into the deep learning network together with the original categorical features.

Through unsupervised learning, we aim to identify groups with similar farming characteristics within each region and convert the region–cluster label into structured information that can be explicitly utilized by the model, thereby providing auxiliary features for the downstream network.

#### 2.3.2. Cyclic Feature Encoding

Pig growth performance is affected by seasonal changes [51]. Although standardized management can reduce production variability to some extent, it cannot completely eliminate the effects of seasonal factors. Because climatic conditions, disease risk, and other factors often vary with the seasons, placement month exhibits a clear periodic pattern. Treating it as a numerical feature may lead the model to incorrectly regard December and January as the most distant months. If it is treated as a categorical feature, the ordinal relationship between months will be lost. Therefore, we use sine–cosine encoding to map placement month into a continuous cyclic representation. Specifically, this module uses trigonometric functions to project the month onto the unit circle, thereby preserving its periodic structure:

(3) $${\mathrm{month}}_{\sin}=\sin\left(\frac{2\pi \times \mathrm{month}}{12}\right),\;{\mathrm{month}}_{\cos}=\cos\left(\frac{2\pi \times \mathrm{month}}{12}\right)$$

The two encoded features, placement_month_sin and placement_month_cos, are then used as new numerical inputs to the network. This encoding method can effectively capture the relationships among different months while ensuring continuity between the end and the beginning of the year. In this way, it preserves the ordinal information of placement month while also representing its periodicity.

#### 2.3.3. Row-Column Fusion Representation

In the input representation stage, we build on the row–column fusion design in [52] and introduce a dual-encoding representation to integrate row-wise and column-wise information. Specifically, the row encoder maps each scalar feature value to a *d*-dimensional vector. For the *j*-th feature value $x_{i,j}$, its row embedding $h_{i,j}^{r}\in R^{d}$ is computed as:

(4) $$h_{i,j}^{r}=\phi_{j}^{r}\left(x_{i,j}\right)=w_{j}x_{i,j}+b_{j}$$

where $w_{j}\in R^{d}$ and $b_{j}\in R^{d}$ are the learnable weight and bias, respectively.

In the column encoding stage, to incorporate global distribution information of each feature column, we construct a column summary ${\tilde{v}}_{j}$. This summary is then mapped to a column embedding $h_{j}^{c}=\phi^{c}\left({\tilde{v}}_{j}\right)$ through a lightweight MLP. Finally, the row representation $h_{i,j}^{r}$ and column representation $h_{j}^{c}$ are fused to obtain the final embedding for the *j*-th feature in sample $x_{i}$:

(5) $$h_{i,j}=h_{i,j}^{r}⊙h_{j}^{c}$$

where ⊙ denotes element-wise multiplication. This fusion enables the final representation to capture both the sample-level value information and feature-level distribution information, which is then fed into the subsequent attention network.

#### 2.3.4. Arithmetic Attention Backbone Network

**Figure 6:**
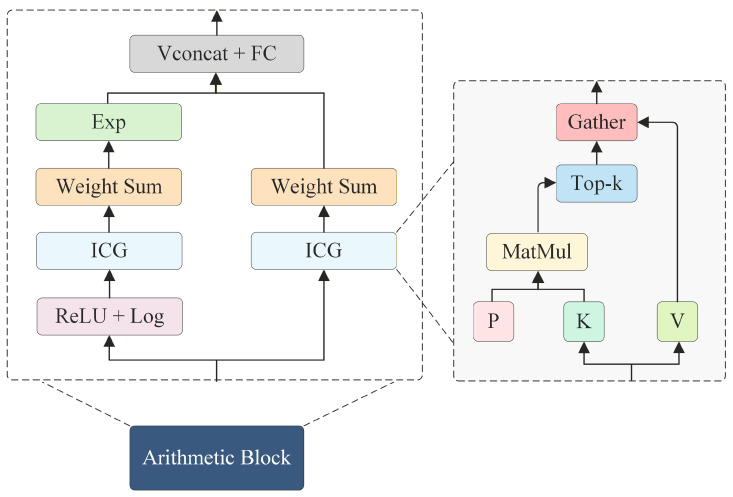
Arithmetic attention mechanism.

Based on the above feature encoding, the numerical and categorical feature embeddings are concatenated along the sequence dimension to form a complete feature sequence for each sample. A learnable CLS embedding is then prepended to the sequence to aggregate global information for the final market weight estimation. The self-attention mechanism in the conventional Transformer architecture mainly models additive interactions among features. To better capture the complex non-linear relationships among indicators in pig production data, we adopt the arithmetic attention mechanism from AMFormer [41] as our backbone network. This mechanism employs parallel additive and multiplicative attention streams to explicitly model both types of arithmetic feature interactions, as shown in Figure 6.

The additive stream follows the classical attention mechanism [39] and computes feature correlations through scaled dot-product attention, thereby extracting additive interaction candidates. Let the input feature embedding matrix be $X\in R^{N\times d}$, where *N* denotes the number of features and *d* denotes the embedding dimension. Query, key, and value matrices are first generated as $Q=XW^{Q}$, $K=XW^{K}$, and $V=XW^{V}$, where $W^{Q},W^{K},W^{V}\in R^{d\times d}$ are learnable parameters. The correlation matrix is then obtained by scaled dot-product attention:

(6) $$A=\frac{QK^{T}}{\sqrt{d}}$$

To accommodate the sparse interaction patterns in tabular data, only the top-*k* most relevant features in each row of the attention scores are retained for normalized weighted aggregation, producing the additive interaction output $O_{\mathrm{add}}\in R^{N\times d}$. This mechanism reduces interference from irrelevant features. Next, the multiplicative stream applies a logarithmic transformation to the input feature embeddings:

(7) $$X_{\log}=\log\left(\mathrm{ReLU}\left(X\right)+1\right)$$

here, the constant term 1 avoids the undefined case of log(0) and allows non-negative production indicators with zero values to be naturally mapped to 0 in log space. Attention is then computed in the log space, and the product semantics are restored through the exponential operator, producing the multiplicative interaction candidate $O_{\mathrm{mult}}$. Through this chained log-attention-exp design, the multiplicative stream explicitly models multiplicative arithmetic interactions, such as placement count × feed consumption, and therefore complements the additive stream. In the fusion stage, the outputs of the two streams are concatenated along the feature dimension and fused by a down-sampling linear layer:

(8) $$O=\mathrm{FC}\left(\left[O_{\mathrm{add}};O_{\mathrm{mult}}\right]\right)\in R^{N\times d}$$

The fused output is then added to the original input through a residual connection and further processed by a feed-forward network (FFN). After multiple stacked layers, the output vector at the CLS embedding position, $z_{\mathrm{cls}}$, is taken, followed by layer normalization and ReLU activation. Finally, a fully connected regression head maps it to the estimated value $\hat{y}$.

### 2.4. Compared Models

To evaluate the effectiveness of the proposed method, we compare RC-AMFormer with 10 strong baselines commonly used for tabular data. These baselines include three tree-based models, namely XGBoost [22], CatBoost [23], and Random Forest [46], five attention-based tabular learning models, namely AutoInt [37], SAINT [38], FT-Transformer [39], ExcelFormer [40], and AMFormer [41], and two classical deep learning models, namely ResNet-like [39] and TabNet [53].

- Tree-based models. XGBoost [22] is an optimized framework based on gradient-boosted decision trees and has strong regularization capability, which is widely used in pig weight estimation. CatBoost [23] is a gradient boosting model specifically designed for categorical features. By using ordered boosting, it alleviates gradient estimation bias and shows clear advantages on mixed-type tabular data. Random Forest [46] is an ensemble learning method that improves generalization ability and robustness by constructing multiple independent decision trees and averaging their predictions or taking a majority vote. It is also one of the most commonly used baseline models for tabular tasks.
- Attention-based models. AutoInt [37] is one of the earliest models to apply self-attention to tabular data, and it captures high-order feature interactions through stacked self-attention layers. SAINT [38] is a tabular-specific architecture that improves learning performance through a hybrid attention mechanism and contrastive pretraining. FT-Transformer [39] adapts the standard Transformer to tabular data and captures high-order feature interactions through contextual embeddings, where its self-attention mainly reflects conventional additive interactions. ExcelFormer [40] introduces semi-permeable attention, interaction attenuation initialization, and tabular-specific data augmentation and can outperform GBDT without hyperparameter tuning. AMFormer [41] is a deep tabular model based on an improved Transformer architecture, whose core design lies in parallel additive and multiplicative attention mechanisms.
- Classical deep learning models. ResNet-like [39] adopts a multilayer perceptron with residual connections, which can alleviate the vanishing gradient problem in deep networks. TabNet [53] employs a sequential attention mechanism and performs feature selection through learnable sparse feature masks. It emphasizes model interpretability and is commonly used in pig weight estimation tasks.

### 2.5. Training Settings

Following [54], we use a sample-wise random split with a fixed random seed to divide the dataset into training, validation, and test sets at a ratio of 64:16:20. Each sample corresponds to a single farming batch. Therefore, no batch appears in more than one subset. At the same time, different batches from the same management unit may be assigned to different subsets. All numerical features are standardized using the mean and standard deviation computed from the training set to ensure a consistent feature scale. All deep learning models are trained using the Adam optimizer, with early stopping based on the validation MSE and a patience of 25. We use the Optuna library [55] for hyperparameter tuning, with validation MSE as the objective metric. The final test performance of each model is evaluated using the hyperparameter configuration that achieves the lowest validation MSE.

For XGBoost, CatBoost, TabNet, AutoInt, and SAINT, we reuse the default settings and hyperparameter search spaces from the comparative experiments reported in [39]. For Random Forest, FT-Transformer, ExcelFormer, AMFormer, and our proposed RC-AMFormer, we refer to the default settings and value ranges in [39,40,41] and construct comparable hyperparameter search spaces to ensure that all models are compared under consistent conditions. All experiments are conducted on a Linux server equipped with an NVIDIA RTX 3080 GPU and CUDA 13.0.

## 3. Results and Discussion

### 3.1. Evaluation Metrics

For the weight estimation task, we use mean squared error (MSE), mean absolute error (MAE), and the coefficient of determination ($R^{2}$) as evaluation metrics. The corresponding formulas are as follows:

(9) $$\mathrm{MSE}=\frac{1}{n}\sum_{i=1}^{n}{\left(y_{i}-{\hat{y}}_{i}\right)}^{2}$$

(10) $$\mathrm{MAE}=\frac{1}{n}\sum_{i=1}^{n}\left|y_{i}-{\hat{y}}_{i}\right|$$

(11) $$R^{2}=1-\frac{\sum_{i=1}^{n}{\left(y_{i}-{\hat{y}}_{i}\right)}^{2}}{\sum_{i=1}^{n}{\left(y_{i}-\bar{y}\right)}^{2}}$$

where *n* is the number of samples in the dataset, $y_{i}$ is the true value, ${\hat{y}}_{i}$ is the predicted value, and $\bar{y}$ represents the mean of the true values.

### 3.2. Estimation Results

In the market weight estimation task, our proposed RC-AMFormer achieves the lowest numerical MSE and MAE among the 10 compared models. As shown in Table 4, its MSE, MAE, and $R^{2}$ reach 11.7667, 2.6353, and 0.6698, respectively. Compared with AMFormer, RC-AMFormer reduces MSE and MAE by 2.27% and 1.22%. These results suggest that the proposed method can provide modest improvements in pig market weight estimation.

The comparison across different models suggests that traditional GBDT models remain competitive in this tabular regression task. XGBoost and CatBoost achieve relatively low errors, which may be related to the ability of tree-based models to capture nonlinear relationships through hierarchical feature-value partitioning. Deep learning models such as TabNet and ResNet-like show performance comparable to some tree-based models. Among attention-based models, FT-Transformer and AMFormer achieve better results than several other neural baselines. This suggests that different attention structures may differ in their ability to model feature interactions in pig farming tabular data.

Unlike most studies that estimate body weight at the individual-pig level using images or point cloud data [56], this study focuses on batch-level market weight concurrent estimation. This setting cannot capture individual differences among pigs, but it can reflect the overall market weight level of a production batch. In addition, the results suggest that non-invasive pig group weight estimation based on routinely accumulated tabular data is feasible to some extent. This data-driven approach may also have potential for extension to other farming scenarios, such as sow body condition monitoring.

### 3.3. Ablation Study

To evaluate the effects of different components, we conduct an ablation study using AMFormer as the baseline, and the results are shown in Table 5. After introducing the regional clustering feature (RC) or cyclic month encoding (Cyclic), the model shows improved performance on the evaluation metrics. Cyclic only re-represents the month feature in a periodic form and introduces minimal additional model complexity, but it still brings certain performance gains. RC also achieves better results than the baseline, suggesting that the discrete structural information derived from region-wise clustering helps characterize latent differences among farming batches.

In contrast, using row-column fusion embedding (Fusion) alone does not bring consistent improvements. This may be because the arithmetic attention mechanism in the backbone already captures part of the feature interactions and relationships among columns [26,52], while Fusion further increases representation complexity. When RC and Cyclic are introduced together, the model shows improved performance over the baseline. After further adding Fusion, all three metrics improve slightly. This suggests that Fusion can provide a modest complementary benefit under the current setting when combined with RC and Cyclic.

We further conduct a clustering feature replacement experiment, in which 2w mortality in the original clustering features is replaced with 5w mortality, while all other experimental settings remain unchanged. As shown in Table 6, the model performs better across all evaluation metrics when two-week mortality is used as the clustering feature.

This result may be related to the different information reflected by the two mortality indicators. 2w mortality corresponds to the early stage after piglet placement, during which pigs face multiple risks, including weaning, transport, environmental change, feed transition, and regrouping [57,58]. At this stage, higher demands are placed on the quality of farm management, and the pigs are relatively more sensitive to them. Therefore, differences in management conditions are more likely to be directly reflected in short-window mortality. At the same time, early management quality affects not only short-term mortality outcomes but may also influence subsequent health status and growth potential [59], thereby affecting final market weight.

In contrast, 5w mortality covers a longer time window. Mortality observed during Weeks 3 to 5 after piglet placement may be affected not only by management practices but also by factors such as regional disease outbreaks and climate conditions. As a result, it may contain more fluctuations unrelated to management differences. By comparison, 2w mortality may retain information more directly related to management quality. In addition, under the current clustering feature combination, some information overlap may exist between 5w mortality and medication cost per pig, which may weaken its complementarity with the other three clustering features.

Overall, 2w mortality and 5w mortality reflect production information from different time windows [49], and their usefulness may vary with the task objective. Under the current task setting, 2w mortality appears to be more suitable as a clustering feature.

### 3.4. Statistical Evaluation of Performance Improvement

To assess the statistical reliability of the observed improvement, we repeat the experiments with 10 different training random seeds while keeping all other experimental settings unchanged.

Table 7 presents the results of the 10 individual runs, and Table 8 summarizes the average performance and statistical significance test results. As shown in Table 8, the average MSE values of AMFormer and RC-AMFormer are 12.0687±0.0902 and 11.8134±0.0867, respectively. A two-sided paired *t*-test on the MSE results suggests that the improvement is statistically significant $\left(p=1.06\times 10^{-6}<0.01\right)$. The two-sided Wilcoxon signed-rank test shows a consistent result $\left(p=1.95\times 10^{-3}<0.01\right)$, providing some statistical support for the observed performance improvement.

### 3.5. Results with Region-Stratified Splitting

To further examine whether the main conclusions are sensitive to the data splitting strategy, we conducted a region-stratified split in addition to the original random split. Specifically, while keeping the same train/validation/test ratio, samples were randomly stratified by region so that the proportion of samples from each region remained approximately consistent across different subsets. This design helps reduce the potential influence of regional distribution imbalance on model evaluation.

As shown in Table 9, the overall performance trend under the region-stratified split is generally consistent with that under the original random split. Compared with the original random split, the overall regression performance under the stratified split is slightly lower. Nevertheless, RC-AMFormer still shows the best overall performance among the compared models, with an MSE of 12.2065, an MAE of 2.6624, and an $R^{2}$ of 0.6611. Since the test set retains a more balanced regional composition, this split may provide a complementary and relatively stricter evaluation perspective. These results suggest that the proposed method maintains a certain level of robustness under different data splitting strategies.

### 3.6. Impact of the Regional Clustering Feature on Tree-Based Models

Table 10 presents the results of introducing the regional clustering feature into tree-based models, which we denote as RC-XGBoost, RC-CatBoost, and RC-RF. The results show that the effect of the regional clustering feature is not fully consistent across different tree-based models. For RC-XGBoost, after introducing the clustering label, the test MSE decreases from 12.2608 to 12.0821. For RC-CatBoost, the MSE decreases from 12.4987 to 12.3457, corresponding to a relative reduction of approximately 1.22%. In addition, both models show improvements in MAE and $R^{2}$.

In contrast, RC-RF does not obtain further improvement after the clustering label is introduced. This may be because Random Forest builds an ensemble through random feature selection and sample resampling across multiple decision trees, and its use of a newly added discrete feature differs from that of gradient-boosted tree models. When the structural information provided by the clustering label is limited or partially overlaps with existing features, its benefit may become unstable. Overall, the regional clustering feature is not consistently effective for all tree-based models. However, the performance improvements observed in XGBoost and CatBoost suggest that this feature can characterize regional structural differences in pig production data to some extent and provide useful complementary information for some tree-based models.

### 3.7. Interpretability Analysis

Based on the estimation results on the test set, we perform SHapley Additive exPlanations (SHAP) attribution analysis to identify the important features influencing market weight, with the tree-based model results used as a reference.

**Figure 7:**
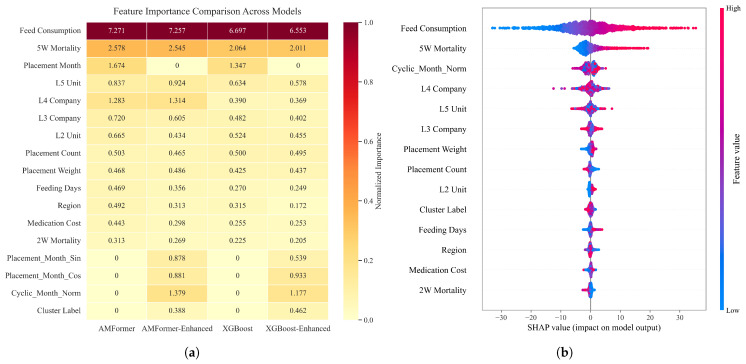
SHAP-based feature importance analysis. (**a**) Feature importance heatmap of AMFormer and XGBoost. The numbers in the cells indicate the raw feature importance values, and darker colors indicate higher relative importance. (**b**) SHAP summary of AMFormer-Enhanced.

Figure 7a shows the feature importance distributions of AMFormer and XGBoost before and after introducing the regional clustering feature and the cyclic encoding of placement month. Figure 7b presents the SHAP distribution of AMFormer-Enhanced, showing how the values of numerical features are associated with the direction of the model output, while categorical features mainly reflect the relative contribution distributions of different categories.

#### 3.7.1. Analysis of Growth, Health, and Seasonal Features

Across the four models, feed consumption consistently ranks first in the SHAP analysis, with importance clearly higher than that of the other features. This finding is consistent with the general understanding in animal nutrition that energy intake is closely associated with growth performance [44].

The importance of 5w mortality consistently ranks second, indicating that herd health in the early stage after placement has an important influence on final market weight. By contrast, 2w mortality shows lower importance, suggesting that mortality measured over different time windows affects the estimation results to different extents. More specifically, 2w mortality mainly reflects health risks during the initial adaptation stage after placement. In comparison, 5w mortality covers a longer growth observation window and more comprehensively characterizes early herd health status and growth stability and therefore shows higher importance in the model. In addition, some of the early risk information reflected by 2w mortality may already be captured by subsequent feed consumption and 5w mortality, which leads to a relatively lower independent contribution in the SHAP analysis.

Among the original features, placement month ranks behind only feed consumption and 5w mortality, indicating that seasonal factors such as temperature and disease pressure still have some influence on pig growth even under modern standardized farming.

After cyclic encoding is introduced, the month feature is represented by two continuous components, placement_month_sin and placement_month_cos. Since these two variables jointly form a two-dimensional representation of month on the unit circle, their contributions are coupled. In addition to calculating the individual SHAP values of the two components, we also calculate the vector magnitude of their SHAP values, namely $\sqrt{{\mathrm{SHAP}}_{\sin}^{2}+{\mathrm{SHAP}}_{\cos}^{2}}$, to measure the overall contribution strength of monthly periodic information. In Figure 7a, this quantity is denoted as Cyclic_Month_Norm. The results show that the vector magnitude reaches 1.177 in XGBoost-Enhanced and 1.379 in AMFormer-Enhanced.

Compared with the original discrete month encoding, cyclic encoding maps the month feature from 12 independent categories into a two-dimensional continuous space. This allows the model to learn the continuity and periodic relationships among months, rather than relying on discrete month values. Such a representation helps capture smooth seasonal variation patterns and reduces dependence on the sample distribution of specific months.

**Figure 8:**
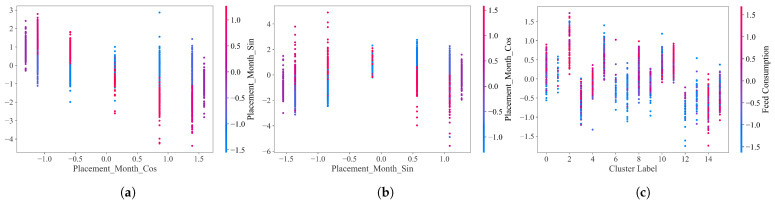
SHAP dependence of selected features. (**a**,**b**) show the interactions between placement_month_sin and placement_month_cos. (**c**) shows the interaction between cluster label and feed consumption.

As shown in Figure 8a,b, the sine and cosine components of month show a clear synergistic effect on the model output. Either component alone cannot fully characterize month information. Their effects are also unevenly distributed across different seasonal windows, indicating that suppressing or promoting effects are more concentrated in certain periods.

#### 3.7.2. Analysis of Hierarchical and Regional Features

Since this study focuses on batch-level market weight concurrent estimation under large-scale standardized pig production, information about the management units to which farms belong should also be considered in addition to growth-related features. The effect-size analysis of hierarchical features in the previous clustering section shows that the explanatory power of hierarchy-related features for market weight generally increases as the management granularity becomes finer (Table 11). Compared with differences at the macro-regional level, more pronounced production differences exist across management units. This provides a basis for incorporating hierarchical information into the model.

Specifically, the $\eta^{2}$ value at the regional level is only 0.032, whereas it reaches 0.145 at the l5 unit level, approximately 4.5 times the effect size of region, as shown in Table 11. Among all adjacent levels, the largest increase in effect size is observed from l3 company to l4 company (+0.039), which is higher than the changes between other levels. This indicates that when the organizational hierarchy is refined from regional management to management units more directly involved in production organization and technical services, more differentiated information related to market weight can be obtained.

From the SHAP results (shown in Figure 7a), the specific rankings of hierarchical variables are not fully consistent across different models. However, l4 company and l5 unit generally maintain relatively high importance and rank only behind feed consumption and 5w mortality in most models. This suggests that fine-grained management units contain rich information about production differences. The importance of hierarchical variables does not show a strictly progressive pattern across levels, which may be related to model structure, feature combinations, and interactions among variables.

To further evaluate the contribution of different hierarchy-related features, we conduct an ablation study on organizational hierarchy features (Table 12). Overall, removing all hierarchy-related features leads to a clear decline in model performance. The MSE increases from 12.0401 to 13.4266, and both MAE and $R^{2}$ also deteriorate, indicating that hierarchical features make a notable contribution to estimation performance. The stepwise removal results further show that removing l5 unit and l4 company, which are closer to the production execution level, causes a relatively larger performance drop. By contrast, the additional impact of removing l3 company is relatively limited, whereas removing l2 unit still leads to a further decline.

This suggests that the information carried by l3 company may be partly covered by l4 company and l5 unit, whereas l2 unit may still provide additional higher-level contextual information. These results are generally consistent with the effect-size analysis and SHAP results, further supporting the effectiveness of fine-grained management information.

From a practical production perspective, higher-level units, namely l2 unit and l3 company, are mainly responsible for macro-level planning and resource allocation. By contrast, lower-level units, such as l4 company and l5 unit, are closer to the actual farming process and are more directly involved in production management, technical services, and farmer guidance. Meanwhile, management-related differences may be more evident at the l4 level, partly explaining its larger impact on model performance. In addition, contract farmers may differ in education level, production skills, and work motivation, which may affect the consistency of production execution [45].

In comparison, the region feature, which represents macro-regional information of farms, generally has lower SHAP values than the organizational hierarchy variables. This may be because the climatic and macro-environmental differences represented by region are further decomposed and absorbed by finer-grained management units. Therefore, although standardized pig production unifies production inputs and farming requirements, it does not fully represent the actual implementation process. Hierarchical variables can reflect differences in farming environment, management conditions, and farmer execution, which may further influence market weight through the pig growth process.

#### 3.7.3. Contribution of Regional Clustering

As shown in Figure 7, the regional clustering feature ranks higher than some original features, capturing information that may not be available in individual variables.

XGBoost tends to be more sensitive to categorical features with clear group boundaries, such as Cluster Label. By contrast, AMFormer tends to capture complex interactions among some categorical variables through joint representation learning (Figure 8c). Although the two models use features in different ways, their results both suggest that regional clustering and cyclic information contribute to market weight estimation to some extent.

#### 3.7.4. Insights into Batch-Level Non-Invasive Weight Estimation

The above analyses show that batch-level market weight estimation is affected not only by direct growth-related factors, such as feed input, herd health, and seasonal variation, but also by production-background factors, such as organizational hierarchy, regional environment, and farmer execution. Unlike studies that estimate pig body weight based on morphological information from images, videos, or 3D point clouds [5,6,7], this study uses production management data naturally accumulated during the farming process for weight estimation, which offers a new direction for batch-level non-invasive weight estimation.

Objective differences remain across farming regions and management units, even under a standardized production system. In this regard, regional clustering and cyclic encoding help the model characterize these underlying differences and their temporal patterns to some extent, improving its ability to capture batch-level heterogeneity in market weight.

### 3.8. Analysis of Regional Farming Patterns

To analyze the structural characteristics within different regions, we perform clustering separately within each region based on all samples and summarize the corresponding regional farming patterns.

**Figure 9:**
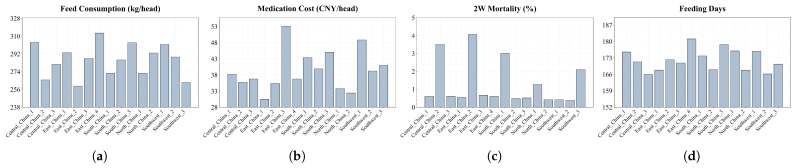
Comparison of farming cluster centers based on four original features. (**a**–**d**) show the farming clusters in different regions in terms of feed consumption, medication cost, 2w mortality rate, and feeding days, respectively.

**Figure 10:**
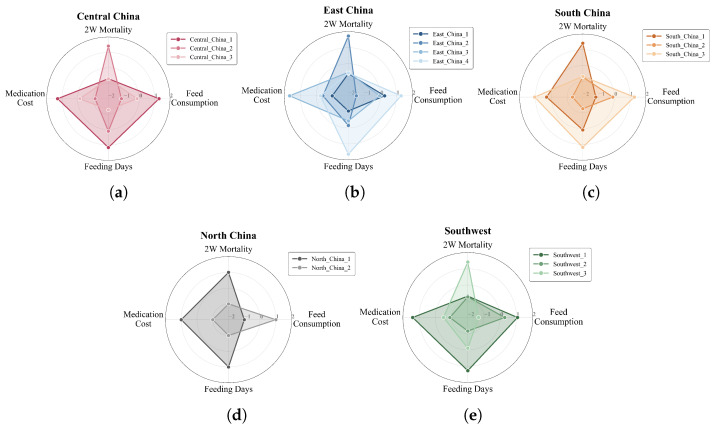
Comparison of standardized cluster centers in the five geographical regions. (**a**–**e**) show the results for Central China, East China, South China, North China, and Southwest, respectively.

**Figure 11:**
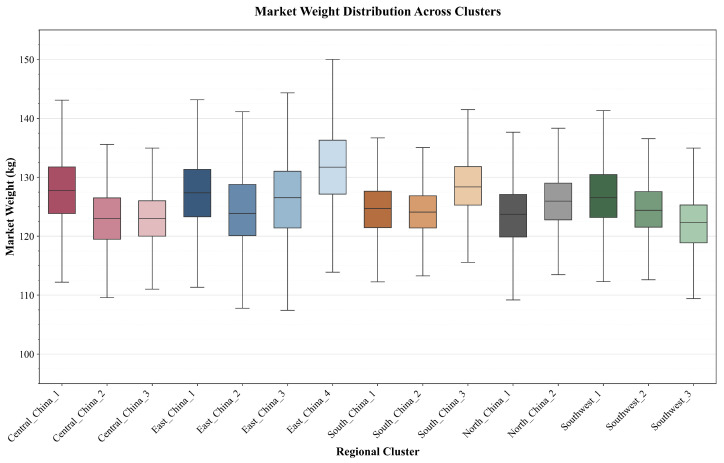
Target average market weight distribution in region–cluster groups.

Table 13 presents the number of batches in each regional cluster, showing clear differences in cluster size across regions. Figure 9 and Figure 10 present the feature differences among cluster centers across the five geographical regions from the perspectives of original feature values and standardized mean values, respectively, and Figure 11 presents the corresponding average market weight distributions. Based on these visualizations, the underlying structure of farming units in different regions was analyzed. Although some continuous transitions exist among regional clusters, the overall patterns can be summarized into three dominant farming modes, namely the long-cycle weight-gain mode, the stable and balanced mode, and the high-risk mode. The representative regional clusters corresponding to these modes are listed in Table 14.

Among the three modes, the long-cycle weight-gain mode is characterized by relatively high feed consumption and long feeding days and is usually associated with higher market weight. Under this mode, pigs generally exhibit a longer feeding cycle, greater feed input, and higher market weight. The stable and balanced mode is characterized by relatively low 2w mortality and medication cost, a relatively short feeding cycle, and an overall moderate level of market weight, reflecting a balance among low risk, short production cycle, and relatively stable output. By contrast, the high-risk mode is characterized by higher 2w mortality and overall lower feed consumption, with relatively low market weight.

It should be noted that the above three modes are primarily a general summary of the regional clustering results and are used to characterize the major structural features in the data rather than to provide a strictly discrete partition of all clusters. Some clusters still show a certain degree of transition or mixture in their feature combinations. For example, although East_China_3 has relatively low 2w mortality, it shows relatively high medication cost, whereas North_China_1 exhibits higher mortality, higher medication cost, and a longer feeding cycle at the same time, reflecting more complex farming characteristics. These characteristics are not completely discrete but instead show a certain degree of continuous variation across different dimensions.

From a production perspective, the three dominant farming modes differ clearly in input level, production risk, and production stability. The long-cycle weight-gain mode is associated with higher market weight but also with greater feed and time input. The stable and balanced mode exhibits better overall production stability. In contrast, the high-risk mode reveals weaknesses in early health management and still leaves room for further improvement. Regarding the cluster distribution (Table 13) representative clusters of the high-risk mode account for a relatively small proportion of samples overall, whereas the stable and balanced mode and some clusters of the long-cycle weight-gain mode cover more batches. This indicates that the high-risk mode is not dominant in actual production and that farming activities still mainly follow relatively stable patterns. However, although high-risk clusters account for only a limited proportion, they are usually associated with higher mortality and greater production fluctuation, and their potential negative impact on overall farming efficiency should not be overlooked.

Because the regional clustering in this study is mainly based on four process-related indicators, the above mode interpretation primarily reflects the major differences along these key production dimensions rather than a complete characterization of the entire farming process.

## 4. Conclusions

Based on more than 35,000 tabular production records, this study proposes RC-AMFormer for batch-level market weight estimation in large-scale pig production. Experimental results on the test set show that RC-AMFormer achieves an MAE, MSE, and $R^{2}$ of 2.64, 11.77, and 0.67, respectively, and shows overall better performance than the ten comparison models. This demonstrate the effectiveness of regional clustering features, cyclic month encoding, and the arithmetic attention structure for weight estimation.

Market weight is influenced not only by growth- and health-related factors but also by regional differences, organizational hierarchy, and seasonal variation. These findings suggest that objective heterogeneity remains across farming units even under a standardized production system.

Several limitations should be noted. The current model is mainly based on batch-level statistical indicators, which makes it difficult to capture individual growth differences. In terms of research scope, this study performs concurrent estimation based on tabular production data accumulated during the farming process, rather than prospective prediction. Therefore, for scenarios requiring prospective prediction several weeks in advance, the temporal availability of input variables should be further constrained. Future studies could further integrate individual-level information, environmental data, and stage-specific production indicators to improve model performance and generalization ability.

Overall, this study demonstrates the feasibility of using tabular data from production management systems for non-invasive body weight estimation. Beyond final average market weight estimation, the proposed data-driven method may be extended to weight estimation at intermediate growth stages. It may also provide a useful reference for large-scale livestock and poultry production with cross-regional and multi-level management structures, as well as for other agricultural production scenarios.

## Figures and Tables

**Table 1:** Indicator descriptions of the pig production data.

Feature	Type	Statistics	Description
Region	Categorical	5 categories	The macro-geographical region to which the farm and pig batch belong, including South China, North China, East China, Central China, and Southwest.
L2 Unit	Categorical	6 categories	A level-2 business management unit under the group headquarters, responsible for coordinating specialized production and operations across business sectors.
L3 Company	Categorical	19 categories	A level-3 regional management company under the level-2 unit, responsible for overall operations in a specific region.
L4 Company	Categorical	96 categories	A level-4 production management company under the level-3 company, directly connected with contract farms.
L5 Unit	Categorical	215 categories	A level-5 grassroots management unit close to front-line farmers, responsible for production services, process tracking, and information recording for pig batches.
Placement Month	Categorical	12 months	The month when piglets in a production batch are placed into the farm.
Placement Count	Numerical	1260.8 ± 766.4	The initial number of piglets at placement.
Placement Weight	Numerical	6.74 ± 0.70 kg	The average initial body weight of piglets at placement.
2W Mortality	Numerical	0.73 ± 1.09%	The proportion of pigs that die or are culled within two weeks after placement, calculated based on the initial placement count.
5W Mortality	Numerical	2.78 ± 2.64%	The proportion of pigs that die or are culled within five weeks after placement, calculated based on the initial placement count.
Feeding Days	Numerical	170.8 ± 6.9	The cumulative number of days from piglet placement to marketing, used to characterize the growth stage and farming progress of the batch.
Medication Cost	Numerical	38.83 ± 12.45 CNY/head	The medication cost per pig accumulated before marketing, calculated based on the initial placement count.
Feed Consumption	Numerical	289.31 ± 22.01 kg/head	An average feed input per pig, calculated from batch-level feed supply records and normalized by the initial placement count.
Market Weight	Target	125.51 ± 5.91 kg	The average body weight of pigs in a production batch at marketing.

**Table 2:** Detailed cleaning rules.

Rule Category	Specific Rules	Removed Records
Basic validity constraints	Feeding Days < 0 or Placement Count < 0	0
Ratio constraints	2W Mortality ∉ [0, 1] or 5W Mortality ∉ [0, 1]	128
Business logic constraints	Market Weight ≤ Placement Weight or 5W Mortality < 2W Mortality	537
Extreme-value thresholds	5W Mortality > 0.5, Feeding Days < 50 or >280, Feed Consumption < 75 or >500 kg/head, Placement Weight ∉ [3, 12.5] kg, Market Weight ∉ [50, 175] kg	142

Some records violate multiple rules, so the category totals exceed the number of unique records removed.

**Table 3:** Regional clustering stability results.

Region	K	Mean ARI	SD
Central China	3	0.9787	0.0133
East China	3	0.9888	0.0069
South China	3	0.9958	0.0038
North China	4	0.9791	0.0112
Southwest	3	0.9668	0.0200

**Table 4:** Performance comparison of different models for pig market weight estimation.

Model	MSE	MAE	$R^{2}$
XGBoost	12.2608	2.6736	0.6560
CatBoost	12.4987	2.6894	0.6493
Random Forest	12.8798	2.7399	0.6386
TabNet	13.9502	2.8357	0.6086
ResNet-like	12.4602	2.7139	0.6503
AutoInt	12.8631	2.7478	0.6390
SAINT	12.8595	2.7450	0.6392
FT-Transformer	12.0846	2.6635	0.6609
ExcelFormer	13.0303	2.7554	0.6344
AMFormer	12.0401	2.6679	0.6622
Ours	**11.7667**	**2.6353**	**0.6698**

Bold values indicate the best performance in each column.

**Table 5:** Ablation study results.

Backbone	RC	Cyclic	Fusion	MSE	MAE	$R^{2}$
**AMFormer**	-	-	-	12.0401	2.6679	0.6622
	√	-	-	11.8963	2.6464	0.6662
	-	√	-	11.8051	2.6402	0.6688
	-	-	√	12.0711	2.6517	0.6613
	√	√	-	11.7785	2.6387	0.6695
	√	√	√	11.7667	2.6353	0.6698

√ indicates that the corresponding setting is included, while “-” indicates that it is not included.

**Table 6:** Comparison of clustering features.

Clustering Feature	MSE	MAE	$R^{2}$
2w mortality	11.8963	2.6464	0.6662
5w mortality	12.0691	2.6624	0.6613

**Table 7:** Performance of AMFormer and RC-AMFormer over 10 random seeds.

Seed ^1^	AMFormer			RC-AMFormer		
	MSE	MAE	$R^{2}$	MSE	MAE	$R^{2}$
30	12.0100	2.6570	0.6630	11.7338	2.6270	0.6708
111	12.2086	2.6748	0.6574	11.9511	2.6461	0.6647
253	12.0542	2.6666	0.6618	11.8397	2.6379	0.6678
367	12.0628	2.6685	0.6615	11.8482	2.6345	0.6676
518	11.9320	2.6597	0.6652	11.6534	2.6292	0.6730
613	12.1396	2.6673	0.6594	11.8392	2.6361	0.6678
678	12.2027	2.6756	0.6576	11.7972	2.6314	0.6690
714	11.9938	2.6624	0.6635	11.8021	2.6381	0.6689
809	12.0659	2.6764	0.6615	11.9151	2.6456	0.6657
955	12.0170	2.6577	0.6628	11.7547	2.6295	0.6702

^1^ The 10 random seeds are randomly sampled from the range 1 to 1000.

**Table 8:** Repeated-run results and significance tests.

Metric	AMFormer	RC-AMFormer	*t*-Test (*p*)	Wilcoxon (*p*)
MSE	12.0687 ± 0.0902	11.8134 ± 0.0867	$1.0628\times 10^{-6}$	$1.9531\times 10^{-3}$
MAE	2.6666 ± 0.0073	2.6355 ± 0.0066	$1.6281\times 10^{-8}$	$1.9531\times 10^{-3}$
$R^{2}$	0.6614 ± 0.0025	0.6685 ± 0.0024	$1.0599\times 10^{-6}$	$1.9531\times 10^{-3}$

**Table 9:** Performance comparison under the region-stratified split.

Model	MSE	MAE	$R^{2}$
XGBoost	12.4020	2.6836	0.6557
CatBoost	12.5855	2.6982	0.6506
Random Forest	12.8962	2.7441	0.6420
TabNet	13.9674	2.8558	0.6122
ResNet-like	12.5929	2.6989	0.6504
AutoInt	13.1136	2.7629	0.6359
SAINT	13.1239	2.7671	0.6357
FT-Transformer	12.3575	2.6765	0.6569
ExcelFormer	13.5360	2.7868	0.6242
AMFormer	12.4380	2.6955	0.6547
Ours	**12.2065**	**2.6624**	**0.6611**

Bold values indicate the best performance in each column.

**Table 10:** Performance of some tree-based models with cluster labels.

Model	MSE	MAE	$R^{2}$
RC-XGBoost	12.0821	2.6596	0.6610
RC-CatBoost	12.3457	2.6841	0.6536
RC-RF	12.9309	2.7443	0.6372

**Table 11:** Effect sizes of regional and organizational hierarchy features.

Feature	$\eta^{2}$	$\Delta \eta^{2}$ vs. Previous	Relative to Region
Region	0.032	–	1.00×
L2 Unit	0.066	+0.034	2.06×
L3 Company	0.095	+0.029	2.97×
L4 Company	0.134	+0.039	4.19×
L5 Unit	0.145	+0.011	4.53×

**Table 12:** Ablation study of organizational hierarchy features.

Model	MSE	MAE	$R^{2}$
AMFormer	12.0401	2.6679	0.6622
*w*/*o* L5	12.4097	2.6807	0.6518
*w*/*o* L5, L4	13.1029	2.7579	0.6324
*w*/*o* L5, L4, L3	13.1561	2.7556	0.6309
*w*/*o* L5, L4, L3, L2	13.4266	2.7942	0.6233

AMFormer denotes the full model, and subsequent rows denote the progressive removal of hierarchy features from l5 unit down to l2 unit.

**Table 13:** Batch counts for clusters in each region.

Region	Cluster	Number of Batches (n, %)
Central China	Central_China_1	1753 (4.9%)
	Central_China_2	608 (1.7%)
	Central_China_3	4054 (11.4%)
East China	East_China_1	2515 (7.1%)
	East_China_2	385 (1.1%)
	East_China_3	1255 (3.5%)
	East_China_4	1298 (3.7%)
South China	South_China_1	793 (2.2%)
	South_China_2	6227 (17.5%)
	South_China_3	3848 (10.8%)
North China	North_China_1	2228 (6.3%)
	North_China_2	3969 (11.2%)
Southwest	Southwest_1	1902 (5.4%)
	Southwest_2	3818 (10.8%)
	Southwest_3	837 (2.4%)

**Table 14:** Dominant farming modes and representative clusters.

Farming Mode	Representative Clusters
Long-cycle weight-gain type	Central_China_1, East_China_4, South_China_3, Southwest_1
Robust and balanced type	Central_China_3, East_China_1, South_China_2, North_China_2, Southwest_2
High-risk type	Central_China_2, East_China_2, South_China_1, Southwest_3

## Data Availability

Restrictions apply to the availability of these data. Data were obtained from Wens Foodstuff Group Co., Ltd. and are available from the corresponding author with the permission of Wens Foodstuff Group Co., Ltd.
